# High-Throughput Phenotyping of Soybean Biomass: Conventional Trait Estimation and Novel Latent Feature Extraction Using UAV Remote Sensing and Deep Learning Models

**DOI:** 10.34133/plantphenomics.0244

**Published:** 2024-09-09

**Authors:** Mashiro Okada, Clément Barras, Yusuke Toda, Kosuke Hamazaki, Yoshihiro Ohmori, Yuji Yamasaki, Hirokazu Takahashi, Hideki Takanashi, Mai Tsuda, Masami Yokota Hirai, Hisashi Tsujimoto, Akito Kaga, Mikio Nakazono, Toru Fujiwara, Hiroyoshi Iwata

**Affiliations:** ^1^ Graduated School of Agricultural and Life Sciences, The University of Tokyo, Tokyo, Japan.; ^2^ Center for Advanced Intelligence Project, RIKEN, Kashiwa, Chiba, Japan.; ^3^ Arid Land Research Center, Tottori University, Tottori, Japan.; ^4^ Graduated School of Bioagricultural Sciences, Nagoya University, Nagoya, Japan.; ^5^ Faculty of Food and Nutritional Sciences, Toyo University, Saitama, Japan.; ^6^ RIKEN Center for Sustainable Resource Science, Yokohama, Japan.; ^7^ Institute of Crop Science, National Agriculture and Food Research Organization, Tsukuba, Japan.

## Abstract

High-throughput phenotyping serves as a framework to reduce chronological costs and accelerate breeding cycles. In this study, we developed models to estimate the phenotypes of biomass-related traits in soybean (*Glycine max*) using unmanned aerial vehicle (UAV) remote sensing and deep learning models. In 2018, a field experiment was conducted using 198 soybean germplasm accessions with known whole-genome sequences under 2 irrigation conditions: drought and control. We used a convolutional neural network (CNN) as a model to estimate the phenotypic values of 5 conventional biomass-related traits: dry weight, main stem length, numbers of nodes and branches, and plant height. We utilized manually measured phenotypes of conventional traits along with RGB images and digital surface models from UAV remote sensing to train our CNN models. The accuracy of the developed models was assessed through 10-fold cross-validation, which demonstrated their ability to accurately estimate the phenotypes of all conventional traits simultaneously. Deep learning enabled us to extract features that exhibited strong correlations with the output (i.e., phenotypes of the target traits) and accurately estimate the values of the features from the input data. We considered the extracted low-dimensional features as phenotypes in the latent space and attempted to annotate them based on the phenotypes of conventional traits. Furthermore, we validated whether these low-dimensional latent features were genetically controlled by assessing the accuracy of genomic predictions. The results revealed the potential utility of these low-dimensional latent features in actual breeding scenarios.

## Introduction

Measuring and modeling plant growth in response to biotic and abiotic stresses is crucial for developing stress-robust cultivation management systems and breeding stress-tolerant varieties [1,2]; however, detailed monitoring of plant traits, such as the number of branches, number of nodes, main stem length, and aboveground weight, presents challenges as the plant grows. Conventionally, these traits have been assessed destructively through plant harvesting, which precludes the continuous monitoring of individual plants. Recently, high-throughput phenotyping (HTP) has emerged as a promising technique for plant growth measurement, offering nondestructive measurement, data analysis, and modeling through machine learning integration [3]. HTP facilitates the estimation of phenotypic values for target traits by applying machine learning models to data collected via nondestructive methods, including imaging and 3-dimensional (3D) measurement of plants. For instance, remote sensing using unmanned aerial vehicles (UAVs) has become a prominent approach to data collection in the field [4,5]. Such UAVs are equipped with various sensors, including RGB cameras [6,7], multispectral cameras [8], and Light Detection and Ranging (LiDAR) devices [9,10]. Although RGB cameras are commonly employed because of their ease of use, affordability, and minimal maintenance requirements, multispectral cameras are becoming popular for measuring vegetation indices associated with growth and yield [11].

The aboveground biomass of plants serves as a critical indicator for assessing the nutritional status and yield potential of crops [12–14]. It represents the total amount of photosynthetic products and thus directly correlates with plant growth, yield, and soil nitrogen levels. In recent years, various models have been proposed to estimate the phenotypic values of the aboveground biomass in soybean (*Glycine max*) by leveraging the relationships between UAV remote sensing images and manually collected data. For instance, Maimaitijiang et al. [15] developed a stepwise multiple regression model to estimate soybean dry weight based on predicted values of plant height and volume, and Yoosefzadeh-Najafabadi et al. [16] utilized 35 vegetation indices obtained via UAV remote sensing to predict soybean fresh weight using ensemble learning techniques such as deep neural network and random forest. Similar models have been developed for other crops, such as wheat (*Triticum aestivum*) [17], rice (*Oryza sativa*) [18], and maize (*Zea mays*) [13,19], which typically require multiple flights or expensive multispectral cameras or LiDAR systems. In contrast, our approach facilitates phenotypic estimation using data from a single UAV equipped with an inexpensive RGB camera.

While conventional machine learning approaches frequently require manual feature engineering to construct models [13,14], deep learning frameworks automatically perform feature engineering based on conventional data [20]. Leveraging this advantage, deep learning has seen widespread adaptation to the HTP of plants over the past decade [21]. Various models have been proposed to evaluate phenotypes in images to address challenges such as object detection, classification, and regression. Object detection models have been used to count the number of flower buds and fruits for yield prediction [22,23], and classification models have been employed to detect plant diseases, determine disease severity, and assess vegetable and fruit quality [24–26]. Regression models have also been used to estimate crop maturity and leaf number [27,28]. Of note, all 3 model types offer more accurate phenotypic measurements than conventional machine learning models.

Plant phenotyping can be conceptualized as the condensation of higher-dimensional information on plant morphology. When visually assessing plant morphology, its multidimensional information is typically condensed into lower-dimensional predefined features. Similarly, hand measurements using destructive methods condense high-dimensional information on plant morphology into lower dimensions, including measurable features, such as plant height and number of branches. HTP, based on images, offers a different approach for condensing high-dimensional information on plant morphology. In this case, high-dimensional information is translated into RGB images or digital surface models (DSMs), from which low-dimensional features are extracted and measured using machine learning models.

Ubbens et al. [3] introduced latent space phenotyping (LSP) as an innovative extension of HTP. This concept involves defining low-dimensional features derived from high-dimensional data, such as plant images captured by sensors and cameras, as phenotypes within a latent space using dimensional reduction methods. These low-dimensional features are then used instead of conventional phenotypes such as agronomic traits [29]. Principal component analysis (PCA) has been utilized extensively for dimension reduction [30–32]. Similar to PCA, LSP is based on the straightforward principle of utilizing low-dimensional features obtained through automatic dimensional reduction methods applied to collected high-dimensional data. However, unlike PCA, it leverages the automatic feature extraction capability of deep learning to extract useful information from highly high-dimensional images and point cloud data and uses this information as a new phenotype. This method is expected to enable the evaluation of phenotypic variations hidden or overlooked by traditional traits rather than merely inferring traditional traits from image and point cloud data. This approach offers greater flexibility than the common HTP methods that typically focus on measuring conventionally defined features. Ubbens et al. [3] utilized low-dimensional features extracted by an embedding network, a type of deep learning model, for a genome-wide association study and quantitative trait locus analysis to identify single-nucleotide polymorphisms (SNPs) and loci associated with drought resistance. Interestingly, they found an overlap in the detected SNPs and loci between LSP and previous studies based on conventionally defined features [3]. Similarly, Gage et al. [33] employed PCA and autoencoder to extract low-dimensional features from 3D data of maize acquired using LiDAR in field settings and analyzed them as plant phenotypes. They also calculated the broad-sense heritability of each low-dimensional feature and conventionally defined hand-measured features, discovering that the low-dimensional features were subject to stronger genetic control than the hand-measured features.

While these previous studies on LSP [3,33] demonstrated the potential of low-dimensional features as selection indices, challenges regarding the interpretability of phenotypes in the latent space were noted as a barrier to their utilization in breeding programs. Ubbens et al. [3] and Gage et al. [33] addressed this issue by employing techniques such as saliency mapping [34] and partial least squares regression to establish connections between conventional descriptors or visible traits and the latent space. In Ubbens et al. [3], saliency maps were used to identify the focus areas of the models from the input data, providing an indirect approach to understanding the latent space. By contrast, in Gage et al. [33], partial least squares regression and its prediction accuracy were used, although this approach may not be universally applicable to all scenarios.

In soybean breeding programs, traits related to aboveground biomass, which is crucial for defining the canopy structure, are key selection targets [34]. Previous studies have focused on developing HTP techniques to estimate phenotypes such as dry weight, canopy coverage rate, canopy volume, and plant height [15,16,35]. Despite this progress, a gap remains in the HTP techniques available for capturing the phenotype of specific traits that determine canopy structure, such as the number of nodes and branches, and the extent of spread. This deficiency has forced breeders and evaluators to rely on manual field measurements, even though many HTP methods are available. To bridge this gap, we employed convolutional neural network (CNN) models [36] to estimate the phenotypes of traits that define the canopy structure. Our approach made use of the simultaneous analysis of ortho-mosaic images and DSMs of the soybean canopy acquired by UAV remote sensing.

Over the past decade, the throughput of phenotyping has been enhanced by the advent of HTP, which has alleviated the burden of labor-intensive and time-consuming tasks. As Ninomiya [20] suggested, HTP not only mitigates the arduous nature of phenotypic data collection but also opens up new avenues for gathering data on traits defined by novel concepts; however, most previous HTP studies focused on measuring traits conventionally defined by evaluators. With the advent of LSP, there is the potential to harness not only the phenotypes of conventional traits measured in breeding programs but also those traits within the latent space that have eluded conventional measurement techniques. Here, we used CNN models to automatically extract low-dimensional features that represent phenotypes within a latent space. To address the challenge of interpretability, we correlated the extracted features with 5 conventional phenotypic descriptors: dry weight, main stem length, numbers of nodes and branches, and plant height. This was achieved by utilizing the weights of the final layer of the CNN model, which provided a link between the conventional descriptors and the latent space. Furthermore, we assessed the genomic prediction [37–40] accuracy of these low-dimensional traits and compared them with conventionally defined traits to determine whether the extracted features could serve as viable selection indicators in actual breeding programs. Genomic prediction is a method for predicting genotype values using genome-wide markers, and these predicted values are used to select superior plants. The accuracy of genomic predictions also serves as a criterion for assessing the heritability of a trait and its potential for genetic improvement. The potential of LSP was evaluated in the studies above.

## Materials and Methods

A field experiment was conducted to validate the effects of drought stress on soybean growth. Soybeans were grown under artificial drought stress and irrigation. The development of each phase was recorded using UAV remote sensing. The phenotypes of the aboveground biomass-related traits were also obtained through destructive investigation. Finally, models were constructed to estimate the phenotypic values of the target traits, which were then utilized as components of the novel LSP method. Further details are presented in the following subsection.

### Field experiment

In 2018, we conducted a field trial for evaluating drought resistance of soybean using a National Agriculture and Food Research Organization core collection consisting of 198 accessions (Table S1) in a sandy-soil experimental field at the Arid Land Research Center, Tottori University, Tottori, Japan (35°32′N latitude, 134°12′E longitude, 14 m above sea level). In the field, the soil was covered with white mulching sheets (Tyvek 700 AG, Dupont, USA) to avoid rainwater soaking the ground and to control soil moisture. Drought-stress and control treatments were established using a drip irrigation system. We here refer to the areas of the control and drought-stress treatments as the control and drought areas, respectively. In the control area, plants were irrigated 3 times per day (0700 to 0900, 1200 to 1400, and 1600 to 1700; 5 h in total). Irrigation was stopped after thinning. Sowing was performed on 2018 July 3, and thinning was performed on 2018 July 20. Fertilizer (15, 6, 20, 11, and 7 g/m^2^ of N, P, K, Mg, and Ca, respectively) was added before sowing. Each plot contained 4 plants of one accession. Each accession was used with 2 replicates in both the control and drought areas, and 792 plots were established. All plants were planted at 20-cm intervals. Details of these plots are shown in Fig. S1.

### UAV remote sensing

We captured images of the field by UAV remote sensing on August 28 before the destructive investigation. A consumer drone (DJI Phantom 4 Advanced, China) was used for UAV remote sensing using RGB images of the field immediately before destructive phenotyping. For UAV remote sensing, the field was divided into 2 parts to address the insufficient battery capacity, and flight plans were set up for each part. The details of each flight plan and shooting conditions for the RGB images were as follows—flight altitude, approximately 12 m; interval for taking pictures, 2 s; focus, autofocus; white balance, automatic; and overlap rate of adjacent pictures (both front and side), 90%. A flight took approximately 15 min and produced 500 to 600 RGB images.

### Measurement of phenotypic values in biomass-related traits

The plant height was measured manually in 2 plants in each plot on 2018 August 21. The dry weight, main stem length, number of nodes, and number of branches of the respective plants were assessed manually through destructive investigation from 2018 September 2 to 5. The length of the main stem length and plant height were defined as the distance from the cotyledonary node to the terminal of the main stem and from the surface of the ground to the highest point of the soybean canopy, respectively. These 5 traits were considered targets for estimation and prediction based on deep learning and genomic prediction. The average phenotypic values of 2 plants in each plot were calculated as the representative phenotypic values of the plot. Normalization was applied to the phenotypic values of all traits by subtracting the mean from the original values and dividing by the standard deviation, resulting in a mean of 0 and a variance of 1. Normalized scores were used as phenotypic values for each plot.

### Image preprocessing

**Fig. 1.**
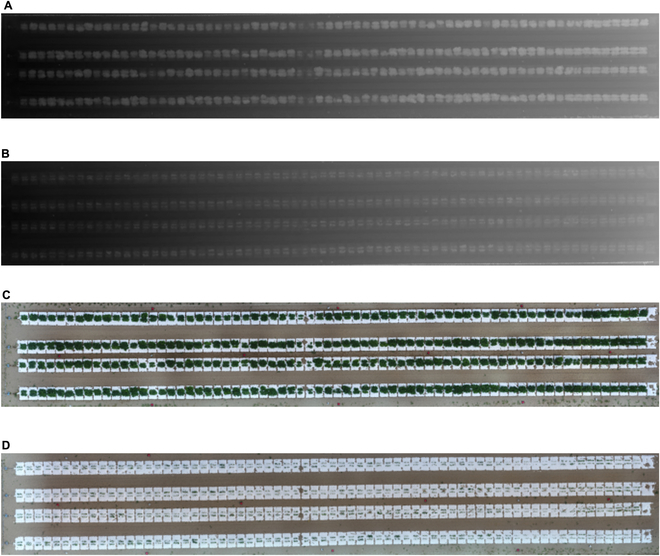
Digital surface model (DSM) and ortho-mosaic images were obtained by remote sensing of soybean field experiment under drought and control conditions. These were constructed using Structure from Motion (SfM) technology with Pix4Dmapper based on images taken from UAV remote sensing. The color intensity of the DSM represents the altitude: the closer to white, the higher the altitude, mainly representing the vegetative canopy; the closer to black, the lower the altitude, representing the ground surface. (A) DSM of control area; (B) DSM of drought area; (C) ortho-mosaic image of the control area; (D) ortho-mosaic image of the drought area.

A DSM and an ortho-mosaic RGB image of the field were obtained from remote sensing images using the software Pix4Dmapper (Pix4D, Switzerland) (Fig. 1). The “3D map” template of Pix4Dmapper was used with slight modifications. While reconstructing the DSM, the “filtering” and “smoothing” options were deactivated to preserve the ups and downs of the plants. The DSM and ortho-mosaic RGB images were segmented for each plot using GPS information. Because nonvegetation regions could introduce noise in the training of deep learning models, vegetation regions in each plot’s DSM and RGB images were extracted as follows. The vegetation and nonvegetation regions were segmented for the RGB image using the Visible-band Difference Vegetation Index (VDVI) [18] with a specified threshold derived through trial and error, which was finally set to 0.057.

$$\text{VDVI}=\frac{2\times G-R-B}{2\times G+R+B}\;$$ (1)

*G*, *R*, and *B* represent the values of green, red, and blue components, respectively. Dark pixels with green component values less than 10 were removed from the vegetation regions, thus producing masked images of vegetation.

Affine transformation was applied to all obtained images to align the position of the canopy and the orientation of the images. Black pixels were added around the vegetation region to ensure the same spatial resolution for the RGB images of all plots. Consequently, images containing only vegetation regions (height: 390 pixels; width: 520 pixels) were obtained (Fig. S2).

For the DSM of each plot, the vegetation regions were extracted based on the positions of the vegetation areas in the ortho-mosaic images of the corresponding plots (Fig. S3). Finally, all preprocessed RGB images and DSMs were manually checked, and images with failed processing results were removed. Images without a canopy in the middle were assumed to be unsuitable for further analysis. The final dataset contained 776 RGB images and DSMs. Image analysis and subsequent deep learning model construction were performed using Python (ver. 3.8.10) (https://www.python.org). OpenCV (ver. 4.4.0.40) (http://opencv.org/) was used for image preprocessing as described above.

To increase the amount of training data for deep learning, data augmentation methods were applied to the ortho-mosaic RGB image and DSM data. The size of the training data obtained from the above procedures was 776, which is smaller than that of the usual applications of deep learning. Because the accuracy of deep learning models generally depends strongly on the size of the training dataset [41], we augmented our dataset. Images were flipped upside down, mirrored, and applied both of them using the *flip* function in OpenCV, which increased the size of the dataset by 4 times its original size.

### Training of deep learning models

To estimate the phenotypic values of the 5 target traits (plant height, dry weight, main stem length, number of nodes, and number of branches), we constructed deep learning models as CNNs using 3 different sets of images: (a) RGB images, (b) DSMs, and (c) RGB images and DSMs as inputs. Below, we refer to these 3 models as RGBNet, DSMNet, and RGB-DSMNet, respectively. All the models consisted of convolutional, maxpooling, and linear layers. Rectified Linear Unit and batch normalization and dropout were adopted as the activation function and methods to avoid overfitting. The number of layers was 14. The structures of all the models and the hyperparameters of each layer are presented in Table S2. All models consisted of 2 main parts: nonlinear feature extraction and linear regression. Each model was aimed at simultaneously estimating the phenotypic values of the 5 target traits. In this process, a 30-dimensional vector was obtained from the nonlinear feature extraction part and then used as an input for a linear regression model estimating the 5 target traits in the regression part. PyTorch (ver. 1.6.0) (https://pytorch.org) was used for model construction.

Below, we introduce mathematical expressions for linear regression. The phenotypic value of the *k*th sample of the *i*th trait was estimated by the following equation (Eq. 2).

$${{\hat{y}}_{i}}^{\left(k\right)}=\sum_{j=1}^{30}w_{\mathrm{ij}}x_{j}^{\left(k\right)}+b_{i}=w_{i}^{T}x^{\left(k\right)}+b_{i}$$ (2)

Here, ${{\hat{y}}_{i}}^{\left(k\right)}\;$ is the estimated phenotypic value of a target trait, *x_j_*^(*k*)^ is the *j*th element of the 30-dimensional vector, which is derived from the last layer of the feature extraction part, *w_ij_* is a weight of *x_j_*^(*k*)^ to estimate the phenotypic value of the *i*th trait, *b_i_* is a bias, **w***_i_* is a vector that has *w_ij_* as elements (**w***_i_* = (*w*_*i*1_, …, *w*_*i*30_)^T^), and **x**^(*k*)^ is a vector that has *x_j_*^(*k*)^ as elements (**x**^(*k*)^ = (*x*_1_^(*k*)^_, …,_ *x*_30_^(*k*)^)^T^), respectively. The values of the 30 elements of *x_j_*^(*k*)^ were scaled such that their means and variances were equal to 0 and 1, respectively. By binding the 5 equations corresponding to the 5 target traits, this equation can be written in matrix form:

$$\hat{Y}=\mathrm{XW}+1b^{T}$$ (3)

Here, $\hat{Y}\;$ is an *n* × 5 matrix of the estimated phenotypic values ${{\hat{y}}_{i}}^{\left(k\right)}$(*n* is the number of samples and 5 is the number of traits), **X** is an *n* × 30 matrix of the extracted features *x_j_*^(*k*)^, **W** is a 30 × 5 matrix of weights, **1** is a vector of length *n* of which are all 1, and **b** is a vector with *b_i_* as elements (**b** = (*b*_1_, …, *b*_5_)^T^). To minimize the difference between the observed value **Y** and the estimated value $\hat{Y}$, the mean square error (MSE) was used as a criterion to determine the optimal values of the regression parameters.

$$\mathrm{MSE}=\frac{1}{n}{\left\|\hat{Y}-Y\right\|}^{2}$$ (4)

Here, $\hat{Y}\;$ is an *n* × 5 matrix of estimated phenotypic values, **Y** is an *n* × 5 matrix of observed phenotypic values, *n* is the number of samples, and ‖·‖^2^ represents the L2 norm. Once the model represented by Eq. 2 was constructed, the estimated phenotypic values of the target traits were calculated using the regression parameters optimized using Eq. 3. The features were extracted from the input images using the feature extraction components of the model. The models were trained under the following conditions: number of epochs: 100; initial learning rate: 0.001; batch size: 128; loss function: mean squared error, and optimizer: Adam [42]. The learning rate was adjusted by the *ReduceLROnPlateau* function in *torch.optim.lr_scheduler* module using the following arguments: factor: 0.5, min_lr: 10^−6^, and patience: 5. In each cross-validation repetition, the best model acquired from the training process was saved and used to estimate the phenotypic values of the 5 target traits. PyTorch was used to train the models and estimate phenotypic values.

The accuracy of all 3 models (RGBNet, DSMNet, and RGB-DSMNet) for estimating phenotypic values **Y** as $\hat{Y}$ was evaluated via 10-fold cross-validation with 20 repetitions. For each cross-validation fold, the dataset was divided into training data (90%) and validation data (10%). In each replicate, the estimation accuracy of the phenotypic values of the 5 target traits (plant height, dry weight, main stem length, number of nodes, and number of branches) was evaluated using the 3 deep learning models (RGBNet, DSMNet, and RGB-DSMNet) and Pearson’s correlation coefficient. Correlation coefficients were calculated for each trait in each fold of cross-validation, and the averages of these values were used for the final evaluation. We also separately calculated the correlation coefficients for each of the 2 irrigation conditions (control and drought) to validate the influence of different growth levels on the estimation accuracy of the deep learning models.

### Feature extraction from RGB images and DSMs based on RGB-DSMNet

The 3 deep learning models estimated the phenotypic values of the 5 target traits in the regression part based on the latent features extracted from the input in the feature extraction part. In other words, each model had a layer consisting of a 30-dimensional vector before the final output layer. We used the values of 30 elements of this vector as latent features. The following analysis was conducted on RGB-DSMNet, which yielded the highest estimation accuracy for most traits, as described in the Results section.

Because there is no fixed rule governing the sequence of elements within the deep learning model layer, the interpretation of each element within the 30-dimensional vector varies with each training instance. For instance, the meaning of a specific element within a 30-dimensional vector varies among the iterations of cross-validation. Consequently, considering each element of the vector as an independent variable could disrupt the analysis, intending to regard them as latent features associated with the 5 target traits. PCA addresses this issue by determining the axes of the latent feature space, which are spanned by the 30 dimensions of the vector, using a variance maximization criterion. The mean and standard deviation values of the proportion of variance of the top 10 principal components in the 10-fold cross-validation with 20 repetitions were calculated to validate the extent to which each principal component illustrated the extracted 30-dimensional vector. Here, the first 5 or 10 principal components were used to summarize the latent features. The relationship between the 30-dimensional vector and the principal component (PC) scores can be represented by Eq. 5:

S=XE (5)

Here, **S** is an *n* × 30 matrix of PC scores for each sample stacked in the row direction, and **E** is a 30 × 30 matrix of the eigenvectors yielding the eigendecomposition of the variance–covariance matrix of **X**. As described above, **X** is an *n* × 30 matrix of the extracted features, and thus, we can calculate the 30 × 30 variance–covariance matrix from **X** because the inverse of **E** is its transpose matrix (**E**^−1^ = **E**^T^):

$$X=SE^{T}$$ (6)

We then visualized the relationship between the PC scores of the 30-dimensional vector and the observed phenotypes of the 5 target traits. Here, the PC scores of the 30-dimensional vector were treated as low-dimensional representations of the RGB images and DSMs. The weight used to derive phenotypic values ($\hat{Y}$) from PC scores (**S**) can be calculated using the weight of the last layer of the RGB-DSMNet (**W**) and the eigenvectors of **X** (**E**) by assigning Eq. 6 to Eq. 3:

$$\hat{Y}-1b^{T}={\mathrm{SE}}^{T}W$$ (7)

Using this equation, **E**^T^**W** was defined as a new weight matrix to derive phenotypic values scaled based on their means from the PC scores. It was defined as **W′** and used in the visualization process. The sign of the weight exhibited a different pattern in each trial of the 10-fold cross-validation with 20 repetitions. Therefore, the most common sign pattern of the weight matrix was adopted to visualize the relationship between the PC scores of the 30-dimensional vector and the observed phenotypes of the 5 target traits. The mean value of **W′** in 10-fold cross-validation through 20 repetitions was calculated and used in visualization.

### Genomic prediction for biomass-related traits and principal components of latent features

Genomic prediction models were built for each PC and irrigation level to evaluate the potential of genomic prediction of PCs with latent features. Genomic prediction is a method used to predict the value of a target trait using genome-wide markers. This study used the Genomic Best Linear Unbiased Predictor (GBLUP) [37–40] as the regression model for making these predictions. While the primary purpose of these predictions is selection, the accuracy of genomic predictions also indicates the degree of genetic control and potential for genetic improvement. Genomic prediction models were constructed for both PCs and conventional traits to determine the prediction accuracy. The following model was used for the genomic prediction of all traits (dry weight, main stem length, number of nodes, number of branches, plant height, and PCs of latent features):

y=1μ+Zg+e (8)

Here, **y** is an *n*-dimensional vector of either the phenotypic values of a target trait or the scores of a PC of the latent features, **1** is an *n*-dimensional vector with all elements 1, *μ* is the grand mean, **Z** is an *n* × *m* design matrix, **g** is an *m*-dimensional vector of breeding values, **e** is an *n*-dimensional vector of residuals, *n* is the number of samples, and *m* is the number of genotypes. We assumed that **g** and **e** followed the multivariate normal distribution, MVN(**0**, **G**σ_g_^2^) and MVN(**0**, **I***_n_*σ_e_^2^), respectively, where **G** is the genomic relation matrix (GRM), σ_g_^2^ is the additive genetic variance, **I***_n_* is an *n* × *n* identity matrix, and σ_e_^2^ is the residual variance. The GRM was calculated based on SNP genotypes obtained from the whole-genome sequencing data of 198 accessions acquired by Kajiya-Kanegae et al. [43]. Only bi-allelic SNPs were used. SNPs were also filtered by minor allele frequency ≥ 0.025 and missing rate < 0.05. Missing entries in the SNP genotype data were imputed using Beagle 5.1 [44]. As a result, genotypes of 4,776,813 SNPs were obtained and were converted to scores: −1 (homozygous for the reference allele), 1 (homozygous for the alternative allele), or 0 (heterozygous for reference and alternative alleles). The GRM was estimated as **G** = **XX**^T^/*c*, where **X** is an *n* × *k* SNP genotype score matrix (*n* and *k* are the numbers of genotypes and SNPs, respectively), and *c* is the normalization constant [45], *c* = 2 Σ_*k*′_ *p*_*k*′_ (1 – *p*_*k*′_), where *p*_*k*′_ is the frequency of the allele at marker *k*′. The accuracy of the genomic prediction model was evaluated using 10-fold cross-validation with 20 repetitions. RAINBOWR package (ver. 0.1.26) [46] was used for the calculations related to genomic prediction.

### Predicting phenotypic values of traits based on principal component scores of latent features

We performed a further genomic prediction to reveal the role of latent features. In this prediction scheme, the phenotypic values of the 5 target traits were calculated based on the PC scores of the latent features predicted from genomic information. Based on Eq. 7, this calculation can be written as

$$\tilde{Y}=\tilde{S}W^{\prime }+1b^{T}$$ (9)

where $\tilde{Y}\;$ is an *n* × 5 matrix of predicted phenotypic values of 5 observed traits for *n* samples, and $\tilde{S}$ is an *n* × *l* matrix of PC scores predicted by genomic prediction. By changing the number of PCs used in prediction (*l*) from 1 to 10, we examined the optimal number of PCs required to predict the genetic variation of each target trait. The number of rows in the weight matrix **W**′ is changed corresponding to *l*. The prediction accuracy was evaluated using the same data splitting as in the cross-validation explained in the previous section. To avoid contamination of the test data during the training process, PCA of the latent features was independently performed in every repetition of the cross-validation. Prediction accuracy was evaluated using Pearson’s correlation coefficient between the predicted and observed phenotypic values for each trait.

## Results

### Nondestructive estimation of target traits using deep learning models

**Fig. 2.**
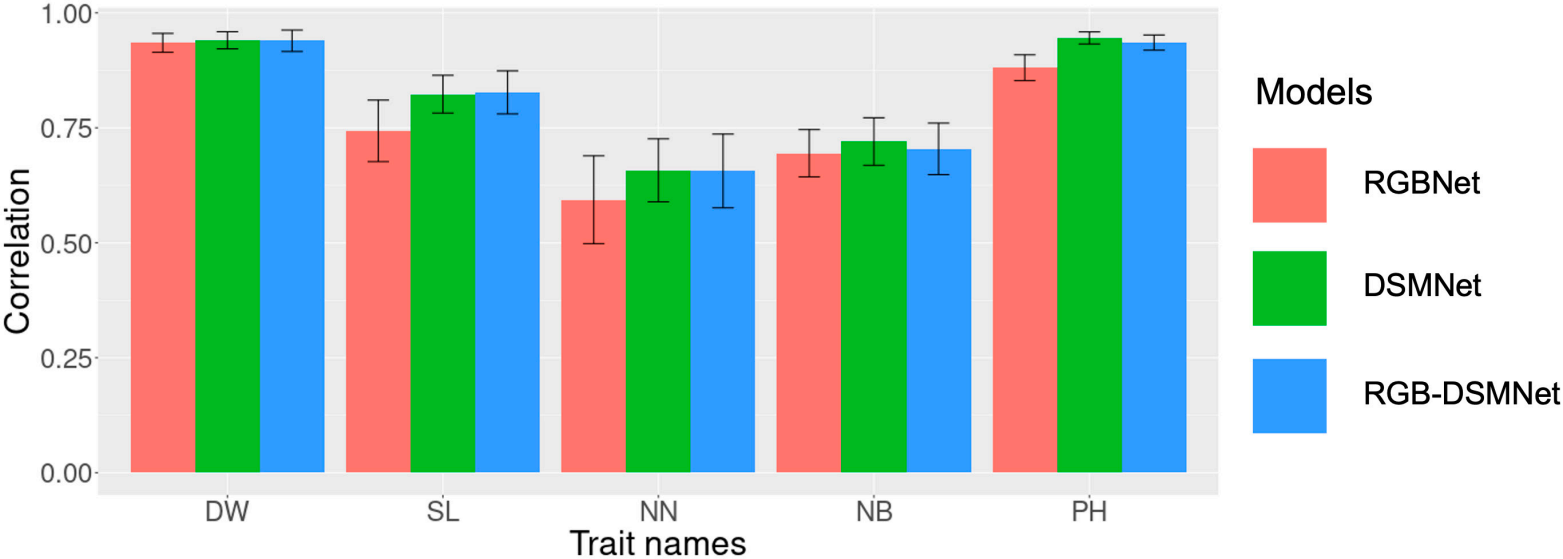
The accuracy of deep learning models to estimate phenotypic values from input data. The models—RGBNet, DSMNet, and RGB-DSMNet—are received RGB images, DSMs, and both of them, respectively. Those data were obtained by UAV remote sensing and image preprocessing by Pix4Dmapper. The bars represent the mean value of the correlation coefficient between observed and estimated values obtained through 10-fold cross-validation with 20 repetitions. The error bars indicate the average standard deviation of the correlation coefficient across the 10-fold cross-validation with 20 repetitions. DW, dry weight; SL, main stem length; NN, number of nodes; NB, number of branches; and PH, plant height.

All 3 models showed moderate to high accuracy for the 5 target traits (Fig. 2). The accuracy of plant height estimation ranged from 0.881 to 0.946, ranking the highest among all traits evaluated. The estimation accuracy of dry weight ranged from 0.935 to 0.940. In contrast, the accuracy of the main stem length, number of nodes, and number of branches estimations was lower, with correlation coefficients ranging from 0.594 to 0.827. RGBNet demonstrated the lowest estimation accuracy among the 3 models, except for dry weight. Conversely, DSMNet provided precise estimates for number of branches and plant height, whereas RGB-DSMNet excelled at estimating dry weight, main stem length, and number of nodes.

**Fig. 3.**
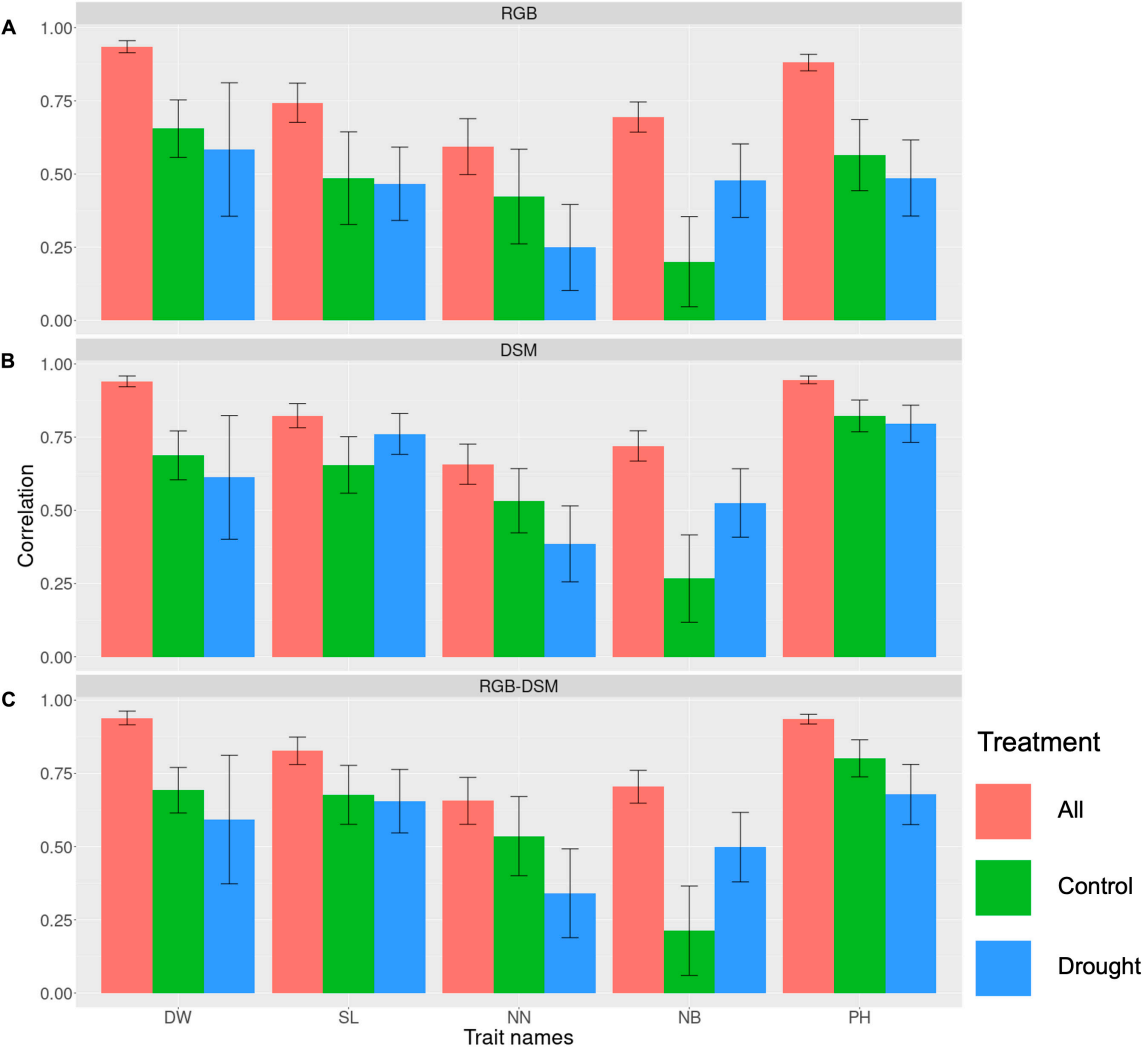
The accuracy of constructed deep learning models to estimate phenotypic values from input data under each treatment. The name of treatment indicates data from which treatment was used to evaluate the estimation accuracy of each model. All, Control, and Drought represent data coming from both control and drought areas, only the control area, and only the drought area, respectively. The bars represent the mean value of the correlation coefficient between observed and estimated values obtained through 10-fold cross-validation with 20 repetitions. The error bars indicate the average standard deviation of the correlation coefficient across the 10-fold cross-validation with 20 repetitions. DW, dry weight; SL, main stem length; NN, number of nodes; NB, number of branches; and PH, plant height. (A) Results of RGBNet. (B) Results of DSMNet. (C) Results of RGB-DSMNet. The name of the models indicates input data. RGBNet, DSMNet, and RGB-DSMNet were received RGB images, DSMs, and both of them, respectively. Those data were obtained by UAV remote sensing, and image preprocessing was obtained by Pix4Dmapper.

We also separately calculated the estimation accuracies of the 5 target traits for each treatment (Fig. 3). In all 3 models (RGBNet, DSMNet, and RGB-DSMNet), the estimation for plants under control conditions was more accurate than that for plants under drought conditions in most cases (except for the estimation results for main stem length by DSMNet and number of branches by all 3 models); however, none of the models could estimate number of branches under control conditions more accurately than under drought conditions.

### Relationships between latent features and biomass-related traits

**Fig. 4.**
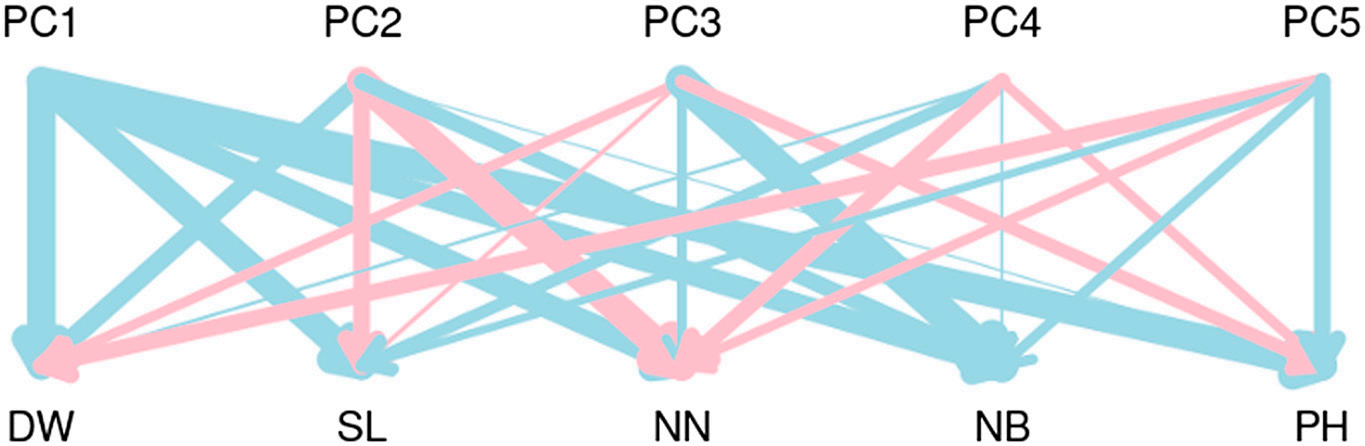
The relationship between principal component of latent features extracted by RGB-DSMNet and conventional traits. The width of lines represents absolute values of weights contained in weight matrix W′ between 2 elements. The wider line indicates that those 2 elements were significantly related. Blue represents that the values of weights are positive, and pink represents that the values of weights are negative. PC, principal component; DW, dry weight; SL, main stem length; NN, number of nodes; NB, number of branches; and PH, plant height.

The relationships between latent features and biomass-related traits are shown in Fig. 4. The relationship between a single trait and the PCs of the latent features varied among the traits. For example, the direction of the effect of the first PC (PC1) on all traits was the same. The direction of effect of PC2 on main stem length and number of nodes was the same, whereas it was opposite for dry weight, number of branches, and plant height, compared with the former traits. Likewise, the direction of effect of PC3 on number of nodes, and number of branches was the same, while it was opposite for dry weight, main stem length, and plant height, compared with the former traits.

### Genomic prediction in biomass-related traits and principal components of latent features

**Fig. 5.**
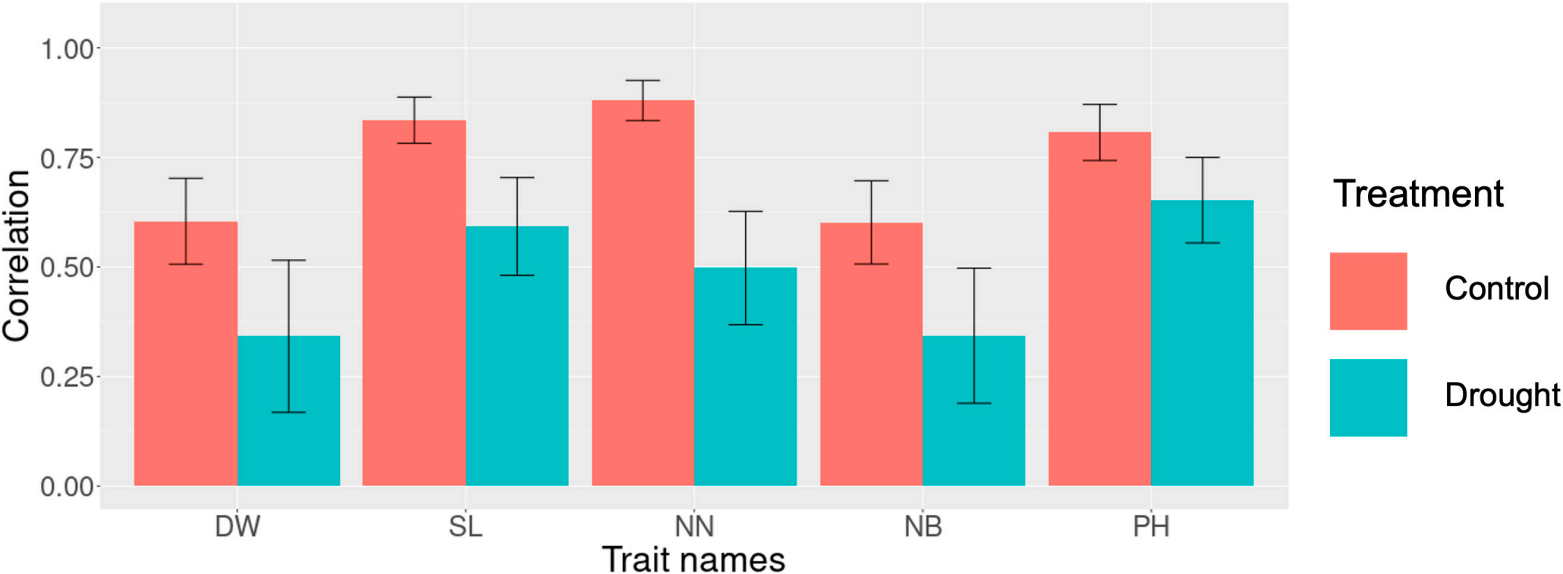
The accuracy of genomic prediction for biomass-related traits. The bars represent the mean value of the correlation coefficient between observed and predicted values obtained through 10-fold cross-validation with 20 repetitions. The error bars indicate the average standard deviation of the correlation coefficient across the 10-fold cross-validation with 20 repetitions. The genomic prediction models were separately constructed for each treatment, such as control and drought conditions, to minimize the effect of environmental factors. Control and Drought indicate that only data from the control and drought area are used, respectively. DW, dry weight; SL, main stem length; NN, number of nodes; NB, number of branches; and PH, plant height.

Genomic prediction models were built for the 5 biomass-related traits using GBLUP. The prediction models showed higher accuracy under control conditions than under drought conditions for all target traits (Fig. 5). The highest accuracy (i.e., correlation coefficient between the predicted and observed values) was 0.880 in number of nodes under control conditions, whereas the lowest was 0.602 in number of branches under control conditions. The highest accuracy was 0.652 in plant height under drought conditions, whereas the lowest was 0.343 in number of branches under drought conditions.

**Fig. 6.**
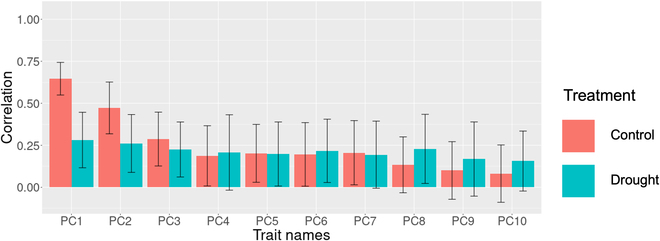
The accuracy of genomic prediction for the principal components of latent features. The bars represent the mean value of the correlation coefficient between observed and predicted values obtained through 10-fold cross-validation with 20 repetitions. The error bars indicate the average standard deviation of the correlation coefficient across the 10-fold cross-validation with 20 repetitions. The genomic prediction models were separately constructed for each treatment, such as control and drought conditions, to minimize the effect of environmental factors. Control and Drought indicate that only data from the control and drought area are used, respectively. PC, principal component.

Genomic prediction models were also built for the PCs of the latent features obtained using RGB-DSMNet (Fig. 6). The prediction accuracy was higher under control conditions than under drought conditions for the first 3 PCs. Under control conditions, the highest accuracy was 0.646 in the 1st PC, while the lowest was 0.081 in the 10th PC. Under drought conditions, the highest accuracy was 0.281 for the 1st PC, and the lowest was 0.156 for the 10th PC.

### Predicting phenotypic values of biomass-related traits based on predicted principal component scores

**Fig. 7.**
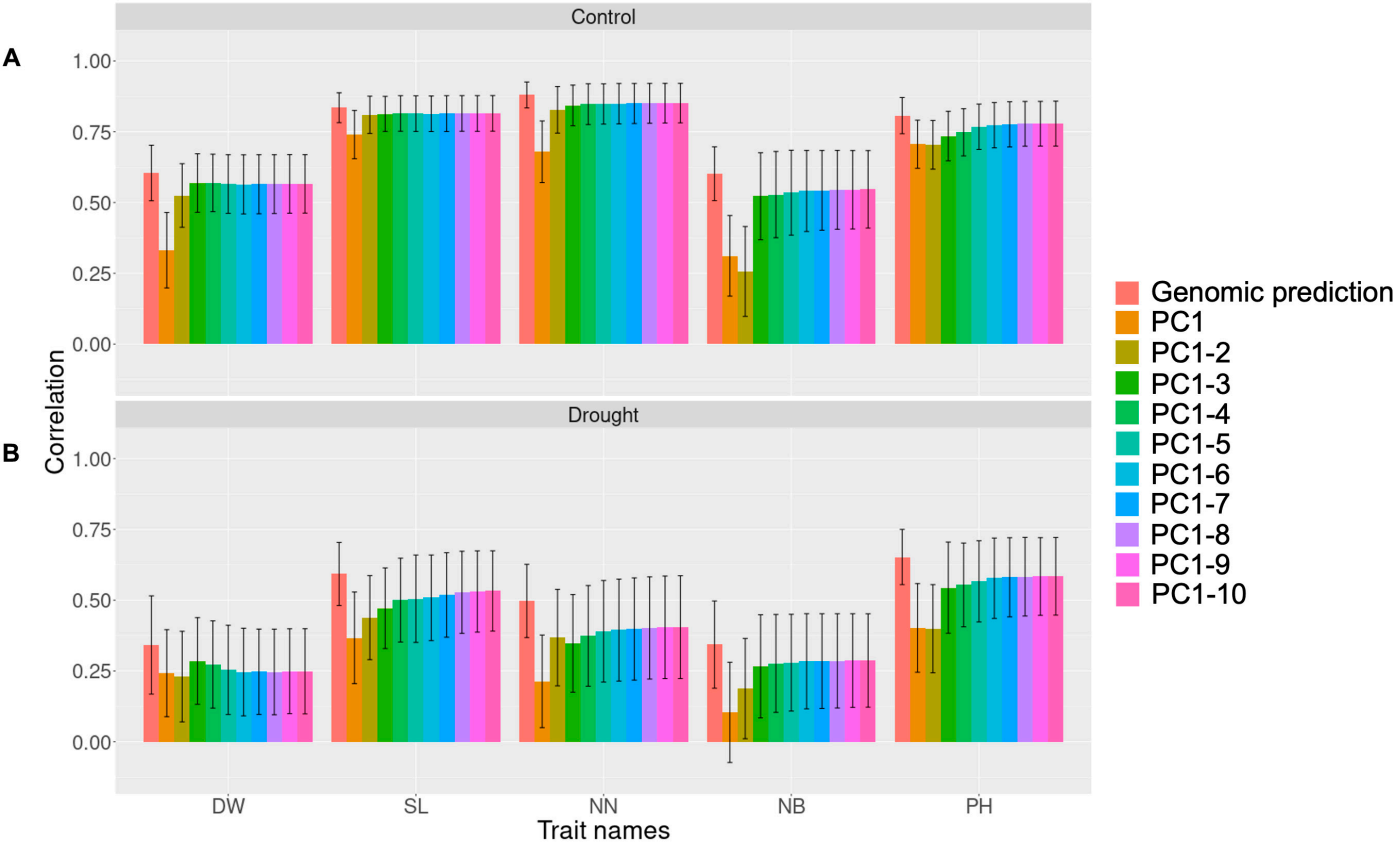
The accuracy of prediction results based on the principal components of latent features predicted by genomic prediction. The bars represent the mean value of the correlation coefficient between observed and predicted values obtained through 10-fold cross-validation with 20 repetitions. The error bars indicate the average standard deviation of the correlation coefficient across the 10-fold cross-validation with 20 repetitions. “Genomic prediction” indicates that the phenotypic values of all traits were directly predicted by GBLUP. “PC1” indicates that the phenotypic values were estimated based on only the principal component score of the first principal component predicted by GBLUP; furthermore, “PC1-n” indicates that the phenotypic values were estimated based on the principal component score of the top *n* principal components predicted by GBLUP, respectively. PC, principal component; DW, dry weight; SL, main stem length; NN, number of nodes; NS, number of stems; and PH, plant height. (A) The results of the control area. (B) The results of the drought area.

The phenotypic values of the 5 target traits were predicted using the predicted scores of the first 10 PCs for genomic prediction. To examine the optimal number of components for prediction, the prediction accuracy was evaluated by increasing the number of PCs (Fig. 7). The results showed that predictions based on the first 2 PCs were as accurate as those based on the first 10 PCs for all traits except number of branches. This suggests that the first 2 PCs contained essential information for estimating the phenotypic values of the 5 target traits; however, in number of branches, an extra PC (i.e., the third PC) seemed necessary. Two different patterns illustrating the relationships between the predicted scores of the PCs and observed phenotypic values of the target traits were confirmed. That is, the first pattern indicates that the first 3 PCs affected those predictions, while the contributions of the others were almost zero or negative (dry weight and main stem length in the control and dry weight in the drought treatment). By contrast, the second pattern suggested a nonnegligible contribution from the fourth and subsequent PCs (number of nodes, number of branches, and plant height in the control and main stem length, number of nodes, number of branches, and plant height in the drought treatment).

## Discussion

In the estimation of the phenotypic values of biomass-related traits, DSMNet exhibited higher accuracy than RGBNet across all traits, suggesting that DSMs contain more essential information related to the traits than RGB images. We anticipated that RGB-DSMNet would achieve higher accuracy than the other 2 models because it utilizes both RGB images and DSMs as inputs; however, while RGB-DSMNet outperformed DSMNet in predicting dry weight, main stem length, and number of nodes, as expected, it showed a lower accuracy in predicting plant height and number of branches than DSMNet (Fig. 2). This finding suggests that RGB images may be redundant when DSMs are available, as the essential data necessary for the prediction of biomass-related traits within RGB images are already contained in the DSMs.

The RGB-DSMNet can estimate the phenotypic values of dry weight and main stem length with high accuracy. Previous studies have developed models to estimate the fresh or dry weight of soybeans because biomass accumulation directly reflects plant growth. Yoosefzadeh-Najafabadi et al. [16] introduced a deep learning model based on a deep neural network to estimate the fresh weight of the aboveground parts of soybean plants by utilizing 35 vegetation indices derived from data acquired by a hyperspectral camera. Although their model outperformed ours in terms of performance metrics, such as the *R*^2^ value, it comes with significant costs for model construction, including a larger number of microplots (approximately 2.5-fold for our field experiment) and genotypes (approximately 25% larger than used here), as well as the use of expensive hyperspectral cameras. Their model utilized specific spectral bands related to chloroplasts and plant pigments as input variables. Incorporating data from specific wavelengths into our models could potentially enhance the performance. Maimaitijiang et al. [15] proposed a stepwise multiple regression model for estimating the dry weight of the aboveground parts of soybean plants, utilizing plant height and volume as architectural data and vegetation indices as spectral data.

A strong correlation between main stem length and plant height may seem intuitive; however, we found that the different models performed best for the destructive estimation of these traits. Specifically, RGB-DSMNet was the top-performing model for main stem length, whereas DSMNet ranked second. Conversely, for PH, DSMNet emerged as the best model, followed by RGB-DSMNet as the second-best model. Regarding the number of nodes, the nondestructive prediction was less accurate, with a correlation coefficient between the observed and estimated values below 0.7. This suggests that capturing the relevant information for a number of nodes via remote sensing from above the plant is challenging.

We successfully extracted phenotypic values of biomass-related traits solely from RGB images and DSMs without requiring feature selection, which is typically needed in other machine learning techniques, such as ridge regression, lasso regression, random forest, and support vector machine [47]. This was achieved by leveraging deep learning (CNN). By employing deep learning, we were able to reduce the time and chronological costs associated with the analysis.

The relationship between the PCs of the latent features and biomass-related traits is illustrated in Fig. 4, revealing several insights. First, PC1 accounted for all biomass-related traits. Second, the direction of the effect of PC2 on dry weight and number of branches was opposite to that on main stem length and number of nodes. This suggests that plants tend to have a long main stem length and large number of nodes, but lower number of branches, shorter plant height, and light dry weight as PC2 increases. In addition, the direction of the effect of PC3 on dry weight and plant height was opposite to that on number of nodes and number of branches. Hence, it was also inferred that plants had lower dry weight, shorter main stem length and plant height, but larger number of nodes and number of branches as PC3 increased. The other PCs exhibited little relationship with the observed traits as their weights were much lower than those of the top 3 PCs.

The relationship between the latent features and target biomass-related traits was assessed by examining the weights of the output layer of the RGB-DSMNet for PC1, PC2, and PC3; however, this relationship was not apparent for the other PCs because of their significantly smaller weight values. The phenotypic values of the target traits were estimated using the latent features predicted by genomic prediction, and their accuracy was evaluated by incrementally increasing the number of components. Consequently, it was suggested that PCs beyond the top 3 PCs were closely associated with subtle changes that could not be captured by the top 3 PCs in the target traits, particularly in the case of plant height under control conditions, and main stem length and number of branches under drought conditions (Fig. 7). It is expected that the role of these subtle changes, as illustrated by the PCs beyond the top 3, will be revealed and discussed in the field of breeding.

In the study by Ubbens et al. [3], LSP was introduced and utilized to explore quantitative trait loci and SNPs associated with specific traits; however, they did not attempt to annotate the meaning of the phenotypes evaluated as LSP using empirical descriptors such as weight, length, and number. In contrast, Gage et al. [33] defined the minimum requirements for utilizing LSP in an actual breeding program, emphasizing that latent features must be annotated by empirical descriptors and genetically controlled. In our study, the latent features were annotated using the weight of the CNN model, and their genetic control was revealed by the genomic prediction results. We did not attempt to compare the performance of our CNN model with those of other models, such as those proposed in previous studies [3,33]. The aims of each study are different, leading to variations in the types and structures of the models according to their specific aims and problem contexts. Our framework acquired the phenotypes of the target traits and annotated the latent features of the plant. Therefore, we strongly anticipate that our technique will introduce a new approach to crop plant breeding.

We investigated the possibility of using the PCs of latent features as new selection indices in breeding fields by comparing the accuracy of genomic prediction for the PCs of latent features with those of conventionally observed biomass-related traits. The accuracy of genomic prediction of the PCs of latent features was generally lower than that of biomass-related traits, with a few exceptions. Notably, under the control conditions, the accuracy of genomic prediction for PC1 was higher than that for dry weight and number of branches, suggesting that PC1 was genetically controlled. The accuracy of the RGB-DSMNet estimates ranged from 0.656 to 0.939 in terms of correlation coefficients, indicating that the network did not capture information about these traits. Additionally, performing the PCA to compare latent features across different folds of cross-validation resulted in information loss (the mean and standard deviation value of the proportion of variance of PC1, PC2, and PC3 in 10-fold cross-validation with 20 repetitions were 47.74%±7.88%, 13.53%±1.64%, and 9.03%±2.17%, respectively, with the remainder being less than 5%). Because of information loss in both RGB-DSMNet and PCA, the accuracy of genomic predictions based on the PCA of latent traits derived from RGB-DSMNet was lower than that of the traits.

As noted by Gage et al. [33], latent features must be related to conventional traits in a visualized manner and genetically controlled for use as selection indices in plant breeding. In our study, PC1 satisfied these conditions, suggesting that PC1 can be used as a new selection index. Furthermore, even if the accuracy of genomic prediction for target traits is low, indicating the low heritability of these traits, the PCs of latent features may contain traits that cannot be conventionally defined. Therefore, depending on the target of the breeding program, it is possible to utilize PCs with low heritability.

The breeding cycle has significantly shortened over the past decade, largely owing to the emergence of genomic selection based on genomic prediction, HTP, and the integration of these 2 techniques [48–50]. Although HTP has made substantial contributions, it frequently requires extensive data storage and time to construct models for phenotyping [20]. By contrast, LSP may offer a faster alternative because it does not always require large datasets or specific models to estimate the phenotype from the data. Consequently, LSP can accelerate the breeding cycle more efficiently than HTP alone. Moreover, the utilization of LSP can reveal features in data that breeders have not previously captured, thereby enhancing the potential of breeding programs [3,20,33].

The methodology proposed in this paper accurately estimates the phenotypes of biomass-related traits in soybeans using data obtained via UAV remote sensing without requiring complex model construction or parameter tuning. A new phenotype was successfully extracted, revealing the relationships between conventional traits by applying deep learning to the collected data. Moreover, these novel phenotypes are genetically controlled, suggesting their potential use as novel selection indices. Based on these findings, our method will enable soybean breeders to select a range of phenotypes derived from a single UAV flight, thereby accelerating breeding cycle. The utilization of LSP may provide breeders with the opportunity to create new lines that cannot be achieved through conventional phenotype-based selection.

## Data Availability

The data supporting the findings of this study are available from the corresponding author, H.I., upon reasonable request.
