# Supplementary figures and images for: High-Throughput Phenotyping of Soybean Biomass: Conventional Trait Estimation and Novel Latent Feature Extraction Using UAV Remote Sensing and Deep Learning Models
Source: Plant Phenomics. 2024 Sep 9;6:0244. doi: 10.34133/plantphenomics.0244 (PMC11382017; doi:10.34133/plantphenomics.0244)

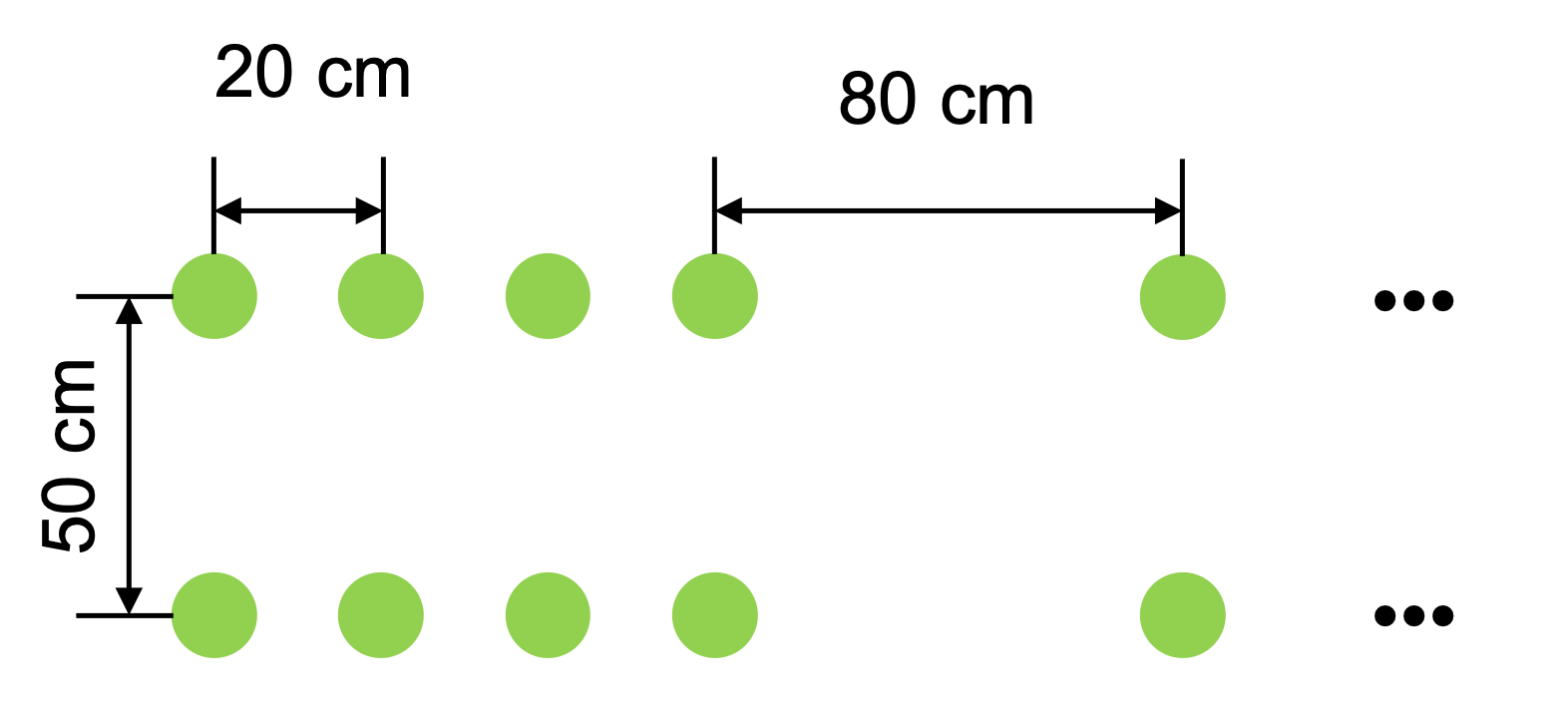

Supplement: Supplementary 1 — Figs. S1 to S3 Tables S1 and S2 [file plantphenomics.0244.f1.zip › FigureS1.png]

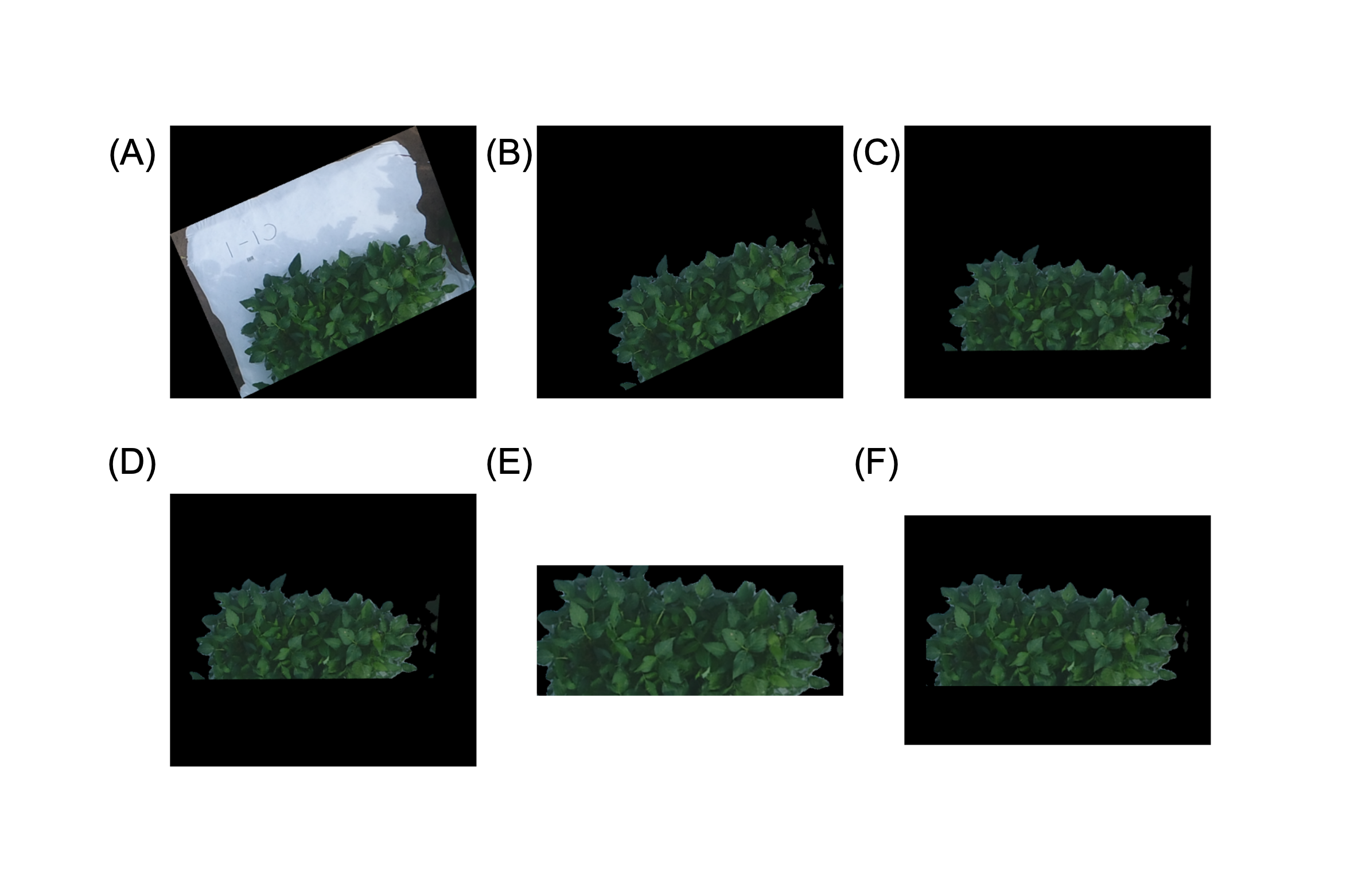

Supplement: Supplementary 1 — Figs. S1 to S3 Tables S1 and S2 [file plantphenomics.0244.f1.zip › FigureS2.png]

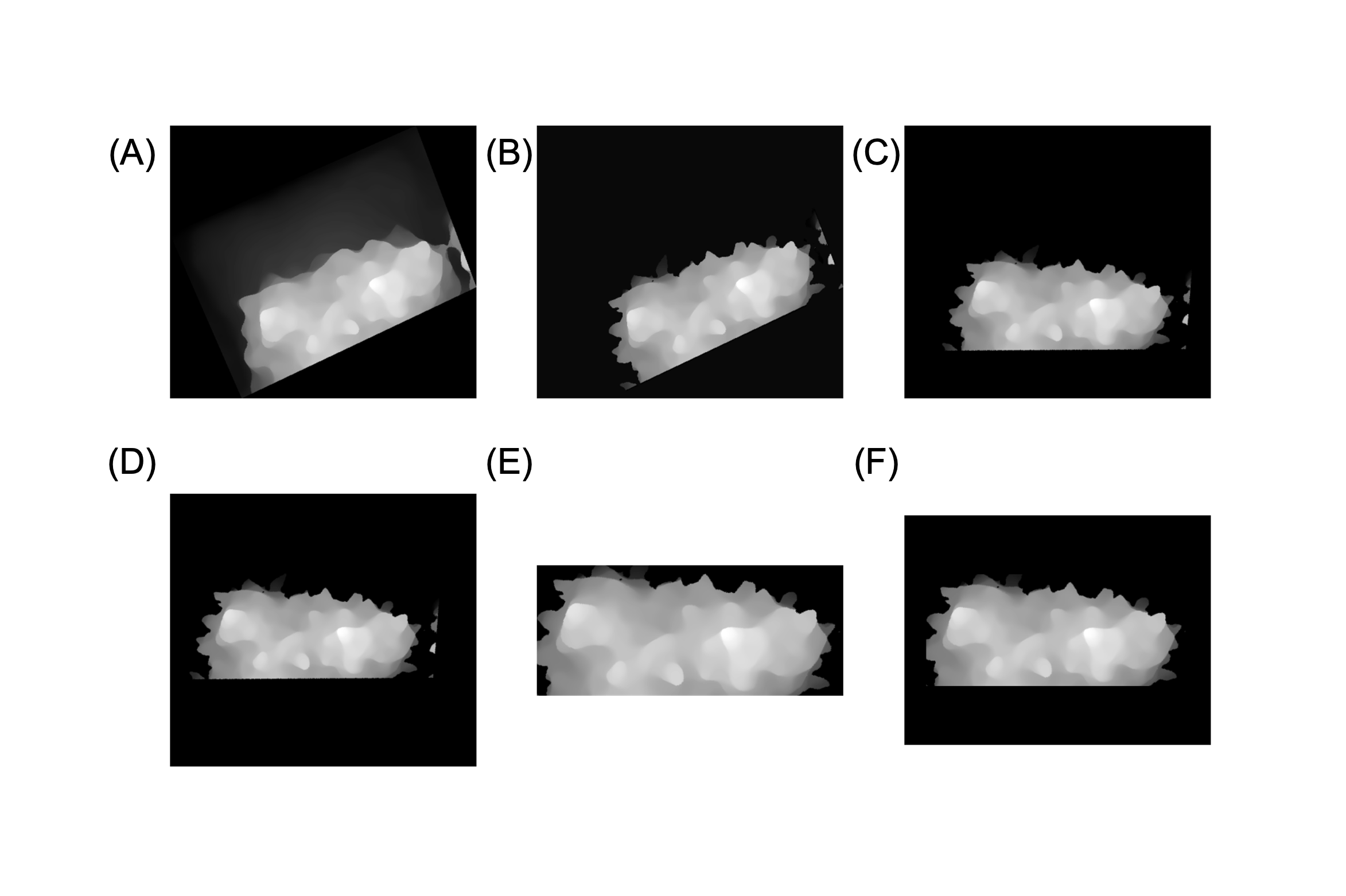

Supplement: Supplementary 1 — Figs. S1 to S3 Tables S1 and S2 [file plantphenomics.0244.f1.zip › FigureS3.png]
